# Supplementary material for: Stochastic force generation in an isometric binary mechanical system
Source: J Gen Physiol. 2024 Nov 19;156(12):e202313493. doi: 10.1085/jgp.202313493 (PMC11577438; doi:10.1085/jgp.202313493)
Supplement: Data S1 — provides the sample python code used for simulations. [file JGP_202313493_DataS1.docx]

Created by Vidya Murthy

@author: vmurthy

class Kinetics_model:

def workingstep(self, number_of_steps: int): #molecules: int):

molecules = int(input('Enter number of molecules: '))

init_molecules_MDP = int(input('Enter number of molecules in MDP: '))

init_molecules_AMD = int(input('Enter number of molecules in AMD: '))

init_molecules_AM = int(input('Enter number of molecules in AM: '))

t_step = 1e-6

kDfo = 250 #s^-1^

kTf = 1e6 #s-1 M-1

kHYDf = 20 #s-1

kHYDr = 2 #s-1

kDr = 0.1 #s-1

kTr = 0.000001 #s-1

displacement = []

energy = []

WS_array = []

t4_mechstep = []

WSr_array = []

t5_mechstep = []

kDf_array = []

t7_ADP = []

disp = []

t8_disp = []

freeenergy = []

t11_dG = []

force = []

t12_force = []

delG = []

t13_dG = []

a_value = []

t15_a = []

a_val = []

t16_a = []

a_Prime_value = []

t17_a = []

a_Prime_val = []

t18_a = []

K = 0.0138

T = 297

x = 0

kfo = 30

kro = 10

ATP = 1e-5

a = 0.5

b = 0.5

k = 0.04

p14 = kHYDr * t_step

p34 = kTf * ATP * t_step

p32 = kDr * t_step

p43 = kTr * t_step

p41 = kHYDf * t_step

mol_state = []

state_count={'MDP': 0, 'AMD': 0, 'AM': 0, 'MT':0}

state_count_2={'MDP':init_molecules_MDP, 'AMD':init_molecules_AMD, ‘AM':init_molecules_AM}

for i in range(number_of_steps):

mol_state.append([])

displacement.append(x)

dGo = -(np.log(kfo/kro))

dGo_Prime = -(np.log(kDfo/kDr))

state_count['MDP'] = mol_state[i].count('MDP')

state_count['AMD'] = mol_state[i].count('AMD')

state_count['AM'] = mol_state[i].count('AM')

state_count['MT'] = mol_state[i].count('MT')

F = k * x #Force genernated at every time step; 5 pN force is generally generated

force.append(F)

t12_force.append(i)

if state_count['MDP'] ==0 and state_count['AMD'] == 0 :

Fo = ((-dGo*molecules)/8 )/4

else:

Fo = ((-dGo*(state_count['MDP'] + state_count['AMD']))/8)/4

if state_count['AMD'] == 0 and state_count['AM'] == 0:

Fo_Prime = ((-dGo_Prime*molecules)/2)/4

else:

Fo_Prime = ((-dGo_Prime*(state_count['AMD'] + state_count['AM']))/2)/4

a1 = F/(Fo)

if a1 < (1/molecules) :

a1 = 1/molecules

a_value.append(a1)

t15_a.append(i)

if len(a_val) >= 0:

if a1 != a_value[i-1]:

a_val.append(a1)

t16_a.append(i)

else:

a_val.append(a1)

t16_a.append(i)

if state_count['MDP'] ==0 or state_count['AMD'] == 0:

d1 = 8

else :

d1 = 8/(a1*(state_count['MDP'] + state_count['AMD']))

if d1 > 8:

d1 = 8

elif a1 >= (1/molecules) and a1 <= 1 :

a1 = F/(Fo)

a_value.append(a1)

t15_a.append(i)

if len(a_val) >= 0:

if a1 != a_value[i-1]:

a_val.append(a1)

t16_a.append(i)

else:

a_val.append(a1)

t16_a.append(i)

if state_count['MDP'] ==0 or state_count['AMD'] == 0:

d1 = 8

else :

d1 = 8/(a1*(state_count['MDP'] + state_count['AMD']))

print('N_AMD+MDP is :', state_count['MDP'] + state_count['AMD'])

if d1 > 8:

d1 = 8

elif a1 > 1:

a1 = 1

a_value.append(a1)

t15_a.append(i)

if len(a_val) >= 0:

if a1 != a_value[i-1]:

a_val.append(a1)

t16_a.append(i)

else:

a_val.append(a1)

t16_a.append(i)

if state_count['MDP'] ==0 or state_count['AMD'] == 0:

d1 = 8

else :

d1 = 8/(a1*(state_count['MDP'] + state_count['AMD']))

if d1 > 8:

d1 = 8

a_Prime = F/(Fo_Prime)

if a_Prime < (1/molecules) :

a_Prime = 1/molecules

# a1 = 0.3

a_Prime_value.append(a_Prime)

t17_a.append(i)

if len(a_Prime_val) >= 0:

if a_Prime != a_Prime_value[i-1]:

a_Prime_val.append(a_Prime)

t18_a.append(i)

else:

a_Prime_val.append(a_Prime)

t18_a.append(i)

if state_count['AM'] ==0 or state_count['AMD'] == 0:

d2 = 2

else :

d2 = 2/(a_Prime*(state_count['AM'] + state_count['AMD']))

if d2 > 2:

d2 = 2

elif a_Prime >= (1/molecules) and a_Prime <= 1 :

a_Prime = F/(Fo_Prime)

a_Prime_value.append(a_Prime)

t17_a.append(i)

if len(a_Prime_val) >= 0:

if a_Prime != a_Prime_value[i-1]:

a_Prime_val.append(a_Prime)

t18_a.append(i)

else:

a_Prime_val.append(a_Prime)

t18_a.append(i)

if state_count['AM'] ==0 or state_count['AMD'] == 0:

d2 = 2

else :

d2 = 2/(a_Prime*(state_count['AM'] + state_count['AMD']))

if d2 > 2:

d2 = 2

elif a_Prime > 1:

a_Prime = 1

a_Prime_value.append(a_Prime)

t17_a.append(i)

if len(a_Prime_val) >= 0:

if a_Prime != a_Prime_value[i-1]:

a_Prime_val.append(a_Prime)

t18_a.append(i)

else:

a_Prime_val.append(a_Prime)

t18_a.append(i)

if state_count['AM'] ==0 or state_count['AMD'] == 0:

d2 = 2

else :

d2 = 2/(a_Prime*(state_count['AM'] + state_count['AMD']))

if d2 > 2:

d2 = 2

else:

d2 = 2/(a_Prime*(state_count['AM'] + state_count['AMD']))

if d2 > 2:

d2 = 2

if state_count['MDP'] == 0 or state_count['AMD'] == 0:

dG = dGo + (np.log(state_count_2['AMD'] / state_count_2['MDP'])) + ((F*d1)/(K*T))

freeenergy.append(dG)

t11_dG.append(i)

if len(delG) >= 0:

if dG != freeenergy[i-1]:

delG.append(dG)

t13_dG.append(i)

else:

delG.append(dG)

t13_dG.append(i)

else:

dG = dGo + (np.log(state_count['AMD'] / state_count['MDP'])) + ((F*d1)/(K*T))

freeenergy.append(dG)

t11_dG.append(i)

if len(delG) >= 0:

if dG != freeenergy[i-1]:

delG.append(dG)

t13_dG.append(i)

else:

delG.append(dG)

t13_dG.append(i)

if x < (L-d1):

e = (1/2) * 0.001* (x**2)

energy.append(e)

kWSf = kfo * np.exp((-1.0)*(a)*(((0.5*0.001*(((x) + d1)**2)) - (0.5*0.001*((x)**2)))/(K*T)))

WS_array.append(kWSf)

t4_mechstep.append(i)

kWSr = kro*np.exp((-1.0)*(1-a)*(((0.5*0.001*((x)**2)) - (0.5*0.001*(((x)+d1)**2)))/(K*T)))

WSr_array.append(kWSr)

t5_mechstep.append(i)

kDf = kDfo * np.exp((-1.0) * (b) * (((0.5 * 0.001 * (((x)+d2)**2)) - (0.5 * 0.001 * ((x)**2))) / (K*T)))

kDf_array.append(kDf)

t7_ADP.append(i)

else:

L > x >= (L-d1)

kWSf = kfo * np.exp((-1.0)* (a) * (((0.5 * 0.001* (L**2)) + (0.5 * k * (d1-L+x)**2)) - (0.5 * 0.001 * (x**2))))

WS_array.append(kWSf)

t4_mechstep.append(i)

kWSr = kro * np.exp((-1.0) * (1-a) * ((0.5*0.001*(x**2)) - ((0.5 * 0.001 * (L**2)) + (0.5 * k * (d1-L+x)))))

WSr_array.append(kWSr)

t5_mechstep.append(i)

kDf = kDfo * np.exp((-1.0) * (b) * (((0.5*0.001 * (L**2)) + (0.5 * k * (d2-L+x)**2)) - (0.5 * 0.001 * (x**2))))

kDf_array.append(kDf)

t7_ADP.append(i)

p12 = kWSf * t_step

p21 = kWSr * t_step

p23 = kDf * t_step

for j in range(molecules):

mol_state[i].append(j)

r1 = np.random.rand()

r2 = np.random.rand()

if i==0:

mol_state[i][j] = 'AM'

self.increment_statecount('AM', state_count)

else:

if mol_state[i-1][j] == 'MDP':

if r1 >= (p12 + p14):

mol_state[i][j] = 'MDP'

elif r2 < (p12 / (p12+p14)):

mol_state[i][j] = 'AMD'

disp.append(x)

t8_disp.append(i)

x = x + d1

disp.append(x)

t8_disp.append(i)

else:

mol_state[i][j] = 'MT'

if mol_state[i-1][j] == 'AMD':

if r1 >= (p21 + p23):

mol_state[i][j] = 'AMD'

elif r2 < (p23 / (p21 + p23)):

mol_state[i][j] = 'AM'

x = x + d2

disp.append(x)

t8_disp.append(i)

else:

mol_state[i][j] = 'MDP'

self.increment_statecount('MDP', state_count)

x = x - d1

disp.append(x)

t8_disp.append(i)

if mol_state[i-1][j] == 'AM' :

if r1 >= (p34 + p32):

mol_state[i][j] = 'AM'

elif r2 < (p34 / (p32 + p34)):

mol_state[i][j] = 'MT'

else:

mol_state[i][j] = 'AMD'

x = x - d2

disp.append(x)

t8_disp.append(i)

if mol_state[i-1][j] == 'MT':

if r1 >= (p41 + p43):

mol_state[i][j] = 'MT'

elif r2 < (p41 / (p41 + p43)):

mol_state[i][j] = 'MDP'

else:

mol_state[i][j] = 'AM'

return

def main():

pass

if __name__ == '__main__':

main()
